# Atypical mucocutaneous manifestations of MPOX: A systematic review

**DOI:** 10.1111/1346-8138.17605

**Published:** 2025-01-03

**Authors:** Andrés Grau‐Echevarría, Daniel Blaya‐Imbernón, Malena Finello, Elena Pérez Zafrilla, Ángel González García, Rodrigo Peñuelas Leal, Carolina Labrandero‐Hoyos, Jorge Magdaleno‐Tapial, Esther Díez‐Recio, Pablo Hernández‐Bel

**Affiliations:** ^1^ Dermatology department of Consorcio Hospital General Universitario de Valencia Valencia Spain; ^2^ Department of Medicine University of Valencia Valencia Spain

**Keywords:** atypical, monkeypox, mpox

## Abstract

MPOX is an orthopoxvirus whose infection has been declared a Public Health Emergency of International Concern in 2022 and 2024. It proved to be a virus with markedly heterogeneous and varied clinical presentation. We performed a systematic PubMed review of articles reporting cases of different clinical manifestations of MPOX until October 2024. The infection has mainly affected men who have sex with men. After 4 to 10 days of incubation, it presents with mucocutaneus lesions and systemic symptoms. Some anatomical sites have shown clinical particularities. Genital edema is a potentially serious complication. The ocular and ear/nose/throat area are other infrequent sites with specific manifestations. MPOX whitlow affects the third finger of the dominant hand and may be associated with extensive inflammation and proximal lymphangitis. Bacterial superinfection is a common complication in the genital area with good response to antibiotic treatment. Immunosuppressed patients may develop severe inflammation and necrosis resulting in poor prognosis. Some authors propose ulceronecrotic MPOX as a defining condition of AIDS. The involvement of women has been exceptional in the current outbreak and has predominantly affected the vulva. Some patients such as healthcare workers, atopics, and people who get tattoos are at risk of developing specific lesions via nonsexual routes. Other atypical manifestations include maculopapular rash and inguinal patch. MPOX is a highly relevant and ongoing infection that can present with multiple atypical manifestations, and the knowledge of which is of great importance to the clinician. We present a unique systematic review of atypical presentations of this infection that may be associated with significant morbidity and mortality, especially in the immunocompromised population.

## 1 INTRODUCTION

MPOX, formerly known as monkeypox, is a double‐stranded DNA orthopoxvirus belonging to the family of *Poxviridae*. Two distinct clades of MPOX virus, clade I (Central Africa) and clade II (West Africa), have existed in different geographical regions and periods. Subclade IIb, identified in the 2022 outbreak, mainly refers to the group of variants widely circulating during the 2022 global epidemic.^1^ It causes an infection with cutaneous and systemic manifestations that has been declared a Public Health Emergency of International Concern in 2022 and 2024.^2^, ^3^ The clinical presentation of MPOX has been shown to be very heterogeneous and varied. In this systematic review of the literature, we describe typical mucocutaneous manifestations and those that elude the usual clinical course of infection (Table S1).

## 2 MATERIALS AND METHODS

The complete protocol for this systematic review was established according to the PRISMA (Preferred Reporting Items for Systematic Review and Meta‐Analysis) guidelines and using the MEDLINE (PubMed) database. The search was limited to manuscripts and reports on human subjects. No language restrictions were applied. The search for studies included articles up to October 1, 2024. We examined the references of included and excluded studies for additional studies. Inclusion criteria were as follows: reported clinical cutaneous or mucosal manifestations that eluded the usual clinical course of the disease with a detailed description of these manifestations. Case reports, cohort studies, clinical trials, and literature reviews were considered for inclusion. Articles lacking an adequate description of clinical manifestations or those not describing atypical manifestations of the disease were excluded. Two authors (AG and PH) independently screened the titles and abstracts of the search results and then the full texts to identify relevant studies for inclusion. Any conflicting decisions were resolved at this stage by discussion. Reference lists of included studies and other review articles were also searched. For the literature search we used the combination of the following keywords: ‘mpox’ or ‘monkeypox’. We did not include ‘cutaneous’ or ‘atypical’ in our strategy to broaden the search results, as we carefully reviewed each article to select those that described an infrequent or atypical manifestation of the disease. Of the 5437 articles found, 136 articles were deemed appropriate for inclusion in this review.

## 3 CLASSIC CLINICAL PRESENTATION

**FIGURE 1:**
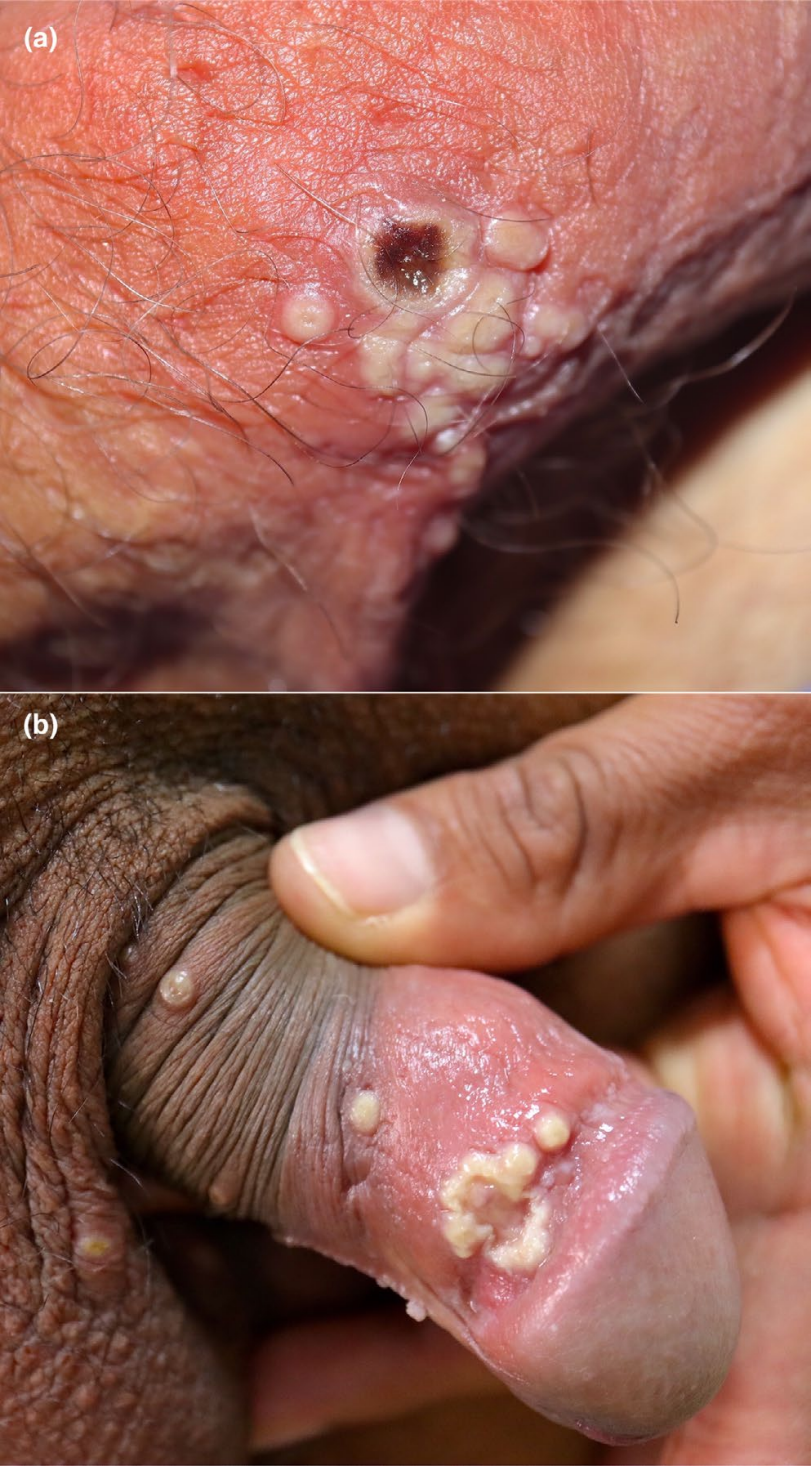
(a) Clustered pseudopustules at the base of the penis. Note the necrotic central ulceration in the larger lesion. (b) On mucosal surfaces, lesions may show a dirty nonspecific appearance.

The incubation period of the infection ranges from 4 to 10 days. More than 90% of patients present with skin manifestations consisting of whitish papules on an erythematous inflammatory base appearing mainly within the area of virus inoculation (in recent outbreaks, such areas coincide with areas involved in sexual contact).^4^ Such erythema may not be noticeable in patients of color.^5^ The lesions have been termed pseudopustules as they are solid lesions containing no purulent material.^6^ Over the next few days, they develop central umbilication with subsequent centrifugally progressive necrosis until the entire lesion is covered (Figure 1a). Eventually, the necrotic crust falls off, leaving residual erythema or a varioliform scar.^7^, ^8^, ^9^, ^10^ Some authors have compared the lesions to a flower in the so‐called Pansy sign.^11^ Ulcers in mucosal areas present with maceration and a dirty background^12^ (Figure 1b).

Previous African endemic cases affected patients of different ages and both sexes, but during the 2022–2024 global outbreak, a dominant sexual route of transmission was identified.^13^ A predilection for men who have sex with men (MSM) has been found, as well as a higher frequency of lesions located in genital and perianal areas, compared with the more monomorphic patterns with truncal predominance and centrifugal spread previously seen in Africa.^14^ Furthermore, infection in the current outbreak has a better prognosis and higher infectivity. The most frequent systemic manifestations include fever, malaise, lymphadenopathy, headache, and arthromyalgia. A nonnegligible percentage of cases have been associated with odynophagia, proctitis, and urethritis. Myocarditis, encephalitis, respiratory, and gastrointestinal disorders have been reported as systemic complications.^15^, ^16^

### 3.1 Solitary lesions

Between 8.5% and 11% of patients present with solitary lesions, most frequently located in the genital area.^17^, ^18^, ^19^, ^20^ This presentation can be a diagnostic challenge and poses a differential diagnosis with other sexually transmitted infections such as syphilis.^21^, ^22^, ^23^, ^24^

## 4 ATYPICAL MANIFESTATIONS IN SPECIAL LOCATIONS

The clinical manifestations of MPOX may vary depending on the route of infection and the stage of the disease. Lesions in the viremic phase tend to be smaller and asymptomatic, whereas lesions at sites of virus inoculation tend to be more inflammatory.^13^, ^25^ Lesions in the current outbreak have prevailed in the genital, perianal, and perioral areas,^26^ in some cases with very florid clinical signs, but there are more anecdotal cases of onset in other locations.

### 4.1 Genital edema

**FIGURE 2:**
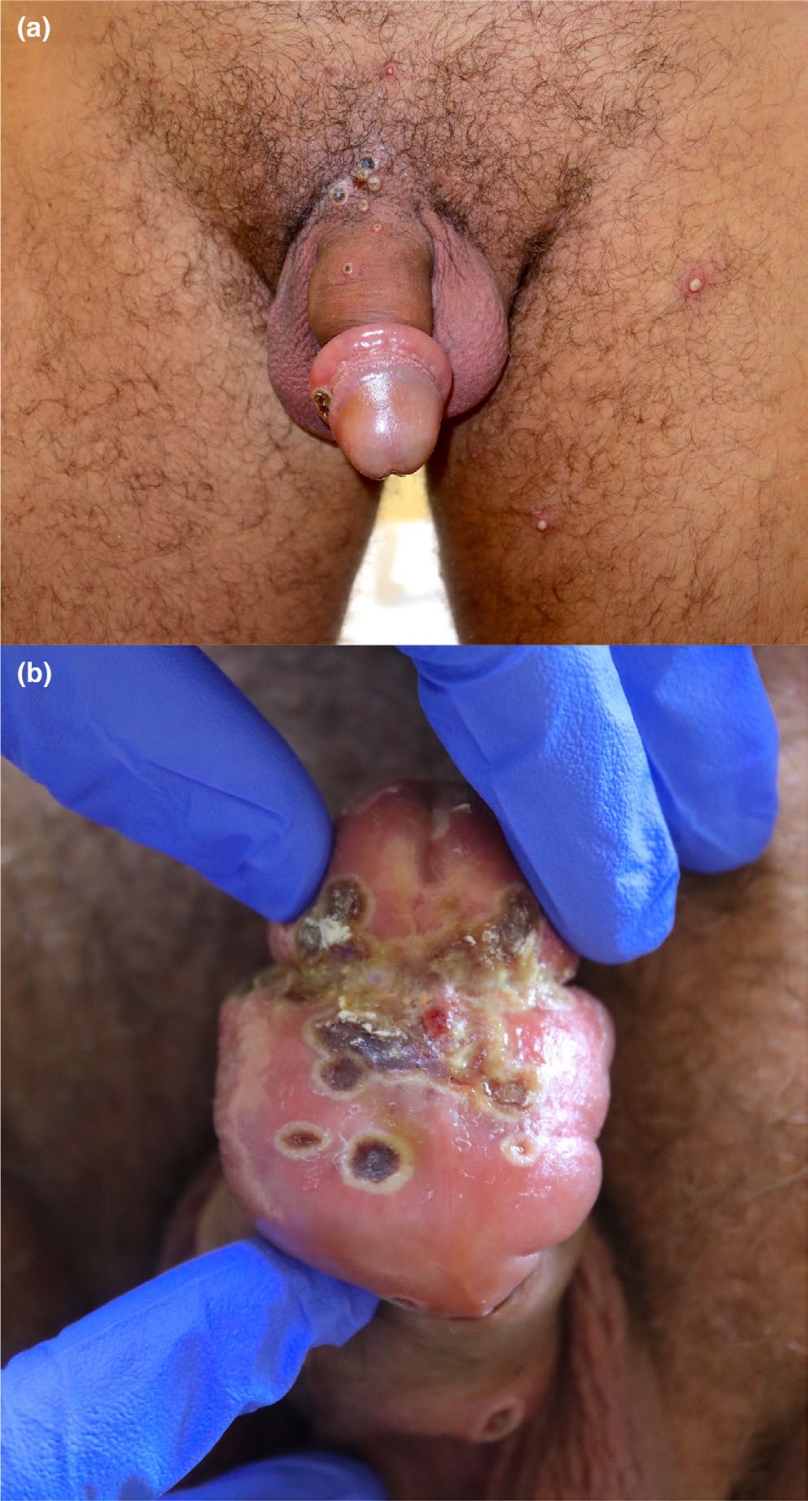
(a) Grouped lesions in the genital region with significant associated edema leading to paraphimosis. (b) Inferior view of the grouped ulcers on the foreskin and the resulting edema.

Genital edema is common in patients with lesions located in this area, particularly if they had large ulcers or clusters of multiple lesions.^27^, ^28^, ^29^ In some cases, it becomes very prominent (Figure 2). Inguinal lymphadenopathy and pain on retraction of the foreskin have been recurrent symptoms. Complications such as phimosis, paraphimosis, and even acute urinary retention have been reported in some patients.^30^, ^31^, ^32^, ^33^, ^34^ Dory flop sign, typical of syphilitic chancre, can be found in patients with MPOX.^35^

Several researchers have linked genital edema to concurrent bacterial cellulitis, although this has not been confirmed by microbiological studies.^36^ Other investigators have described similar cases that do not respond to various antibiotic treatments and with negative culture results.^37^ These cases cast doubt on the theory of bacterial superinfection as a cause of edema and raise the possibility that it is caused by lymphatic obstruction due to regional lymphadenopathy.^38^

Most patients show improvement with the use of local treatment and anti‐inflammatory drugs, both steroidal and nonsteroidal. Only in very rare cases, when lesions are extensive and show severe necrosis, is aggressive surgical debridement including subcutaneous cellular tissue required.^39^, ^40^ This condition resembles Fournier gangrene, without purulent discharge or malodor.^41^

### 4.2 ENT area

**FIGURE 3:**
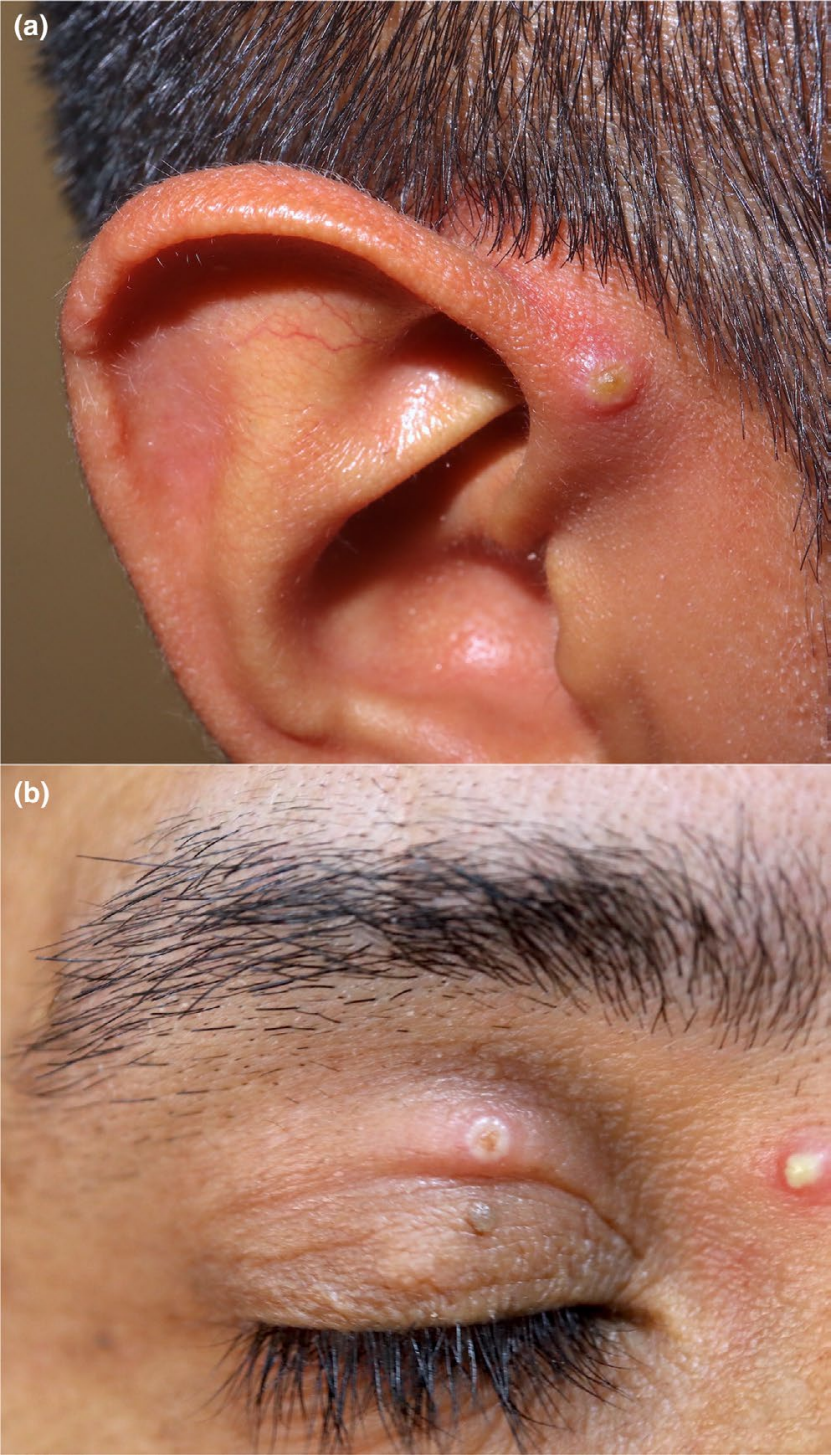
(a) Pseudopustule at the root of the helix. (b) Pseudopustules on the eyelid and glabella in a patient with HIV and eyelash hypertrichosis. In these cases, typical skin lesions may be the clue to the diagnosis of ophthalmological or otorhinolaryngological presentations.

Ear, nose, and throat manifestations have been present in some patients with MPOX. They have generally been milder than in pre‐2022 endemic cases. Some of these include cough, oral ulcers, odynophagia, otalgia, tonsillitis, and tonsillar and laryngeal ulcers. These nonspecific manifestations require a high index of suspicion, and the presence of typical cutaneous findings may be the key to diagnosis^42^, ^43^, ^44^, ^45^ (Figure 3a).

The pinna is a rare site for this type of infection. Some cases have been described with typical pseudopustules associated with perichondritis and marked erythema and edema in the earlobe. This complication could be considered similar to facial or genital edema, as the lobule is also an area of very loose subcutaneous cellular tissue. Some of these patients had previous eczema or received a piercing as a possible route of contagion.^46^, ^47^, ^48^

Nasal lesions have been particularly complicated. In patients with lesions in the nose, additional bacterial infections, severe necrosis, and edema surrounding the lesions have been documented.^49^, ^50^ An additional challenge lies in the fact that this region has great aesthetic relevance, and the sequelae can considerably affect quality of life. The use of antivirals, either topical or systemic, could mitigate symptoms and reduce possible sequelae.^51^

### 4.3 Ocular lesions

Ophthalmological manifestations are rare and include pseudopustules on the periocular skin, blepharitis, conjunctivitis, scleritis, corneal ulcers and keratitis, uveitis, and even corneal scarring and blindness.^52^, ^53^, ^54^, ^55^, ^56^ Cases have been reported with significant swelling in this location comparable to edema in the genital area^57^ and even ocular necrosis.^58^ The presence of pseudopustules on the eyelids may be a diagnostic clue as some cases do not display lesions on the rest of the integument^59^ (Figure 3b). Considering the possible sequelae of blindness in cases of corneal involvement, patients with suspected ocular affectation should be evaluated by an ophthalmology specialist.^60^

### 4.4 Acral lesions

**FIGURE 4:**
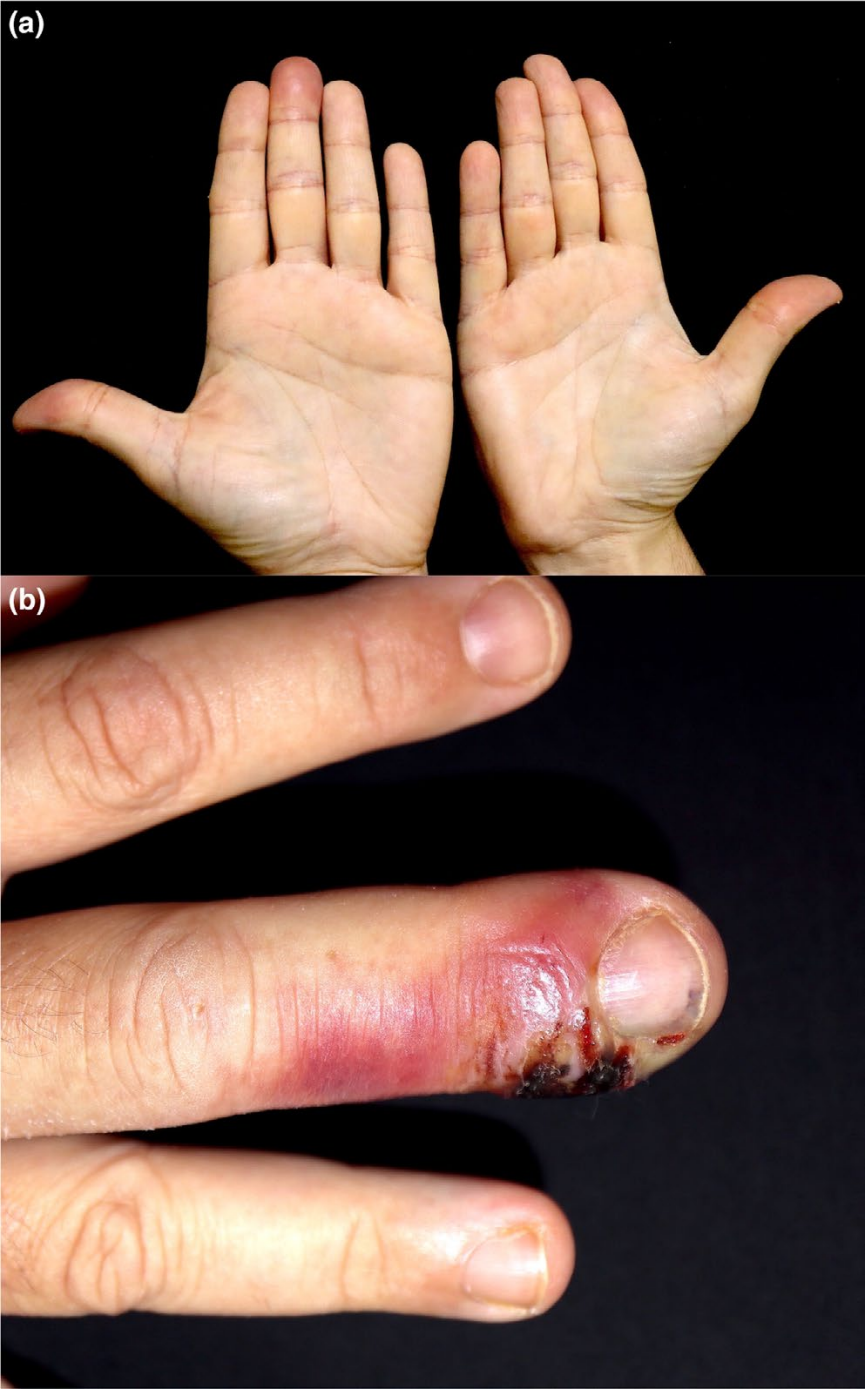
(a) A left‐handed patient with erythema in the thumb of the third finger of the dominant hand. (b) Periungual inflammation in a patient with MPOX whitlow.

MPOX whitlow is an uncommon manifestation that was first identified during the 2022 and 2023 outbreaks. Some researchers suggest that local exposure to the virus during anal‐digital insertion may be a possible mechanism of transmission. This justifies the predominant involvement of the third finger of the dominant hand (Figure 4). It should be noted that this type of localization is common in other sexually transmitted infections affecting the fingers.^61^ MPOX whitlow should be distinguished from other similar conditions, which include bacterial superinfection in acral lesions, for which culture and, in case of doubt, empirical antibiotics are recommended.^10^

Acral lesions on the dorsum of the hand with extensive edema and erythema have been described in poorly controlled HIV‐positive patients. In severe cases, they can lead to extension and necrosis of the lesions over the entire hand.^62^ Similarly, cases involving the sole of the foot have shown further extension to the dorsum of the foot with abundant associated necrotic tissue.^63^ In cases with such extensive and destructive lesions, poorly controlled HIV infection must be ruled out.

Lymphangitis has been reported as an extremely rare manifestation in acral lesions. In some patients, it was identified as bacterial lymphangitis, which responded well to antibiotic treatment. In other cases, after ruling out bacterial infection, the virus was considered the causative agent.^64^

## 5 BACTERIAL SUPERINFECTION

Lesions caused by MPOX often progress to necrosis and ulceration, which increases the risk of bacterial superinfection. Cases of cellulitis and abscesses associated with MPOX, with or without microbiological identification of the additional pathogen, and which respond positively to empirical antibiotic therapy, have been documented.^65^, ^66^, ^67^, ^68^ Some studies describe these complications as rare and mild in severity. The genital area is the most affected, accounting for up to 80% of cases, and requiring intravenous antibiotic therapy or surgical management in <3% of patients.^68^ However, not all perilesional inflammation in the context of MPOX is attributable to bacterial superinfection.^61^ Although the virus seems to facilitate the entry of secondary infections, it can also provoke its own inflammatory reactions in cases of extensive or area‐specific lesions.

## 6 ATYPICAL MANIFESTATIONS IN SPECIAL POPULATIONS

### 6.1 Immunosuppressed patients

In immunocompromised patients, especially those with poorly controlled HIV, high viral loads, and low CD4 cell counts, lesions can be much larger and widespread.^69^, ^70^, ^71^, ^72^, ^73^ The severity of the disease, as well as the risk of hospitalization, complications, and mortality, are directly related to the patient's immune status, being more severe in those with a lower CD4 count per mm^3^ and higher viral loads.^74^, ^75^, ^76^, ^77^, ^78^, ^79^, ^80^, ^81^

These lesions have shown a slower course, with a prolonged resolution time.^82^, ^83^, ^84^ Some patients develop monomorphous lesions that increase in size and number over several weeks, acquiring a necrotic and hemorrhagic appearance.^85^ This type of presentation may be associated with symptoms such as diarrhea, pneumonia, and other systemic complications, as well as typical manifestations of self‐limiting MPOX, such as proctitis, pharyngitis, odynophagia, and even genital edema. Disseminated and necrotic forms of MPOX have been proposed as a defining condition of AIDS.^86^, ^87^, ^88^ A severe necrotizing form of mpox with systemic manifestations has been described. This clinical form may occur in individuals with CD4 cell counts <200 cells per mm^3^ and is associated with high mortality (≈15%).^86^ Presentations such as hemorrhagic MPOX, reminiscent of hemorrhagic smallpox, have also been observed, exhibiting purpuric and petechial lesions paralleling the clinical course.^89^ Exophytic and verrucous lesions,^90^, ^91^, ^92^, ^93^ large ulcers mimicking pyoderma gangrenosum,^94^ and pseudo‐tumors with friable tissue that differ markedly from typical whitish papules progressing to central necrosis^95^ have been observed.

In this group of patients, systemic manifestations are much more common, including ocular involvement, endocarditis, pneumonitis, and central nervous system involvement.^96^ Bacterial superinfection in extensive necrotic lesions has frequently led to sepsis and multiorgan failure.^86^

Some of these patients died, but others responded well to treatment with tecovirimat and appropriate management of their HIV infection.^90^ Nevertheless, cases of recurrence and resistance to tecovirimat have been reported.^97^ Several authors warn about possible worsening with the introduction of antiretroviral therapy in patients with HIV who have severe immunosuppression. This immune reconstitution inflammatory syndrome can affect up to 25% of patients and prove fatal in a high percentage of cases.^86^

Other causes of immunosuppression, such as solid organ transplantation, lymphoproliferative syndromes, and chemotherapy, may also increase the risk of developing an aggressive course superimposable to that described in patients with poorly controlled HIV.^69^, ^98^, ^99^, ^100^, ^101^

### 6.2 Women and children

Female cases accounted for <3% of this outbreak, with the vulva being the most affected area.^102^, ^103^ The presentation was similar to that seen in men with whitish papules on an erythematous base with delayed umbilication and necrosis of the center. However, the course has been milder with fewer lesions and systemic symptoms, and HIV coinfection was significantly less frequent.^104^ In some cases, they may extend to the cervix with the characteristic whitish rim surrounding areas of necrosis.^105^ Cases in pregnant women were rare during the 2022 outbreak,^106^ but they were reported in previously endemic cases and in the recent 2024 outbreaks in Africa. These cases were often associated with frequent obstetric complications and significant fetal mortality.^107^ Infections in infants were very rare during the current outbreak and have generally had a good prognosis.^108^, ^109^ In neonates, tecovirimat has been used with good tolerance.^110^

### 6.3 Healthcare workers and tattoos

Nosocomial transmission, although infrequent, is of particular relevance among healthcare workers. Contact isolation is mandatory during patient care, and, if not properly followed, or in case of accidents such as needle punctures, infection can occur without sexual contact. In these settings, postexposure prophylaxis may be useful to reduce the severity of the disease.^111^ Clinical manifestations in these cases usually include larger and earlier lesions at the site of inoculation, followed by fever and systemic symptoms. Generalized skin lesions tend to be smaller and milder, probably related to viremia. The clinical picture often develops following a needle stick in the hand of staff while taking samples from an infected patient.^64^, ^111^, ^112^

Another route of transmission has involved tattoo, microblading, and piercing parlors.^113^ Outbreaks involving several patients over a period of weeks have been traced. In these cases, a high proportion of women were affected, with a notable appearance of locoregional lymphadenopathy around day 7, before the onset of skin symptoms.^114^ The lesions started in the area of the tattoo and then spread.^115^, ^116^

### 6.4 Eczema monkeypoxicum

Eczema monkeypoxicum was first described during the recent outbreak in 2022. It is a rare complication that mainly affects patients with atopic dermatitis caused by disruption of the skin barrier, with similar pathogenesis to eczema herpeticum or coxsackium, in which the virus invades the eczema lesions. Clusters of lesions are present, and perilesional edema may be a key sign to identify them. This rash may be accompanied by locoregional lymphadenopathy and systemic symptoms, in addition to the characteristic skin lesions in eczema‐affected areas. Generalized papules following viremia may be the diagnostic clue. Unlike the typical presentation of MPOX, sexual contact does not seem to be involved in these cases, which usually occur after occupational exposure or without a clear history of exposure.^48^, ^117^

### 6.5 Vaccinated patients and reinfection

The presence of MPOX has been detected by serological evidence and PCR‐positive samples in asymptomatic patients.^118^, ^119^ This is much more frequent among patients vaccinated with the modified vaccinia Ankara vaccine.^120^ In addition, the possibility of reinfection with the virus has been increasingly reported,^121^, ^122^ with similar severity but a shorter duration.^123^ Screening and vaccination strategies play a vital role in shortening the chain of transmission and mitigating the severity of infection.

### 6.6 Scars

Information on scarring is scarce in the current literature. Recent studies indicate an elevated risk of 40% to 50% for scarring in patients with MPOX.^124^, ^125^ The risk has been related to clinical onset with skin manifestations as well as the presence of superinfection and abscesses.^124^, ^126^ Depressed and atrophic scars are frequently seen, but hypertrophic scars and areas of scarring alopecia in the perioral area have been identified.^127^ A case of a patient with generalized anetoderma after infection has also been documented.^128^

## 7 OTHER ATYPICAL MANIFESTATIONS

**FIGURE 5:**
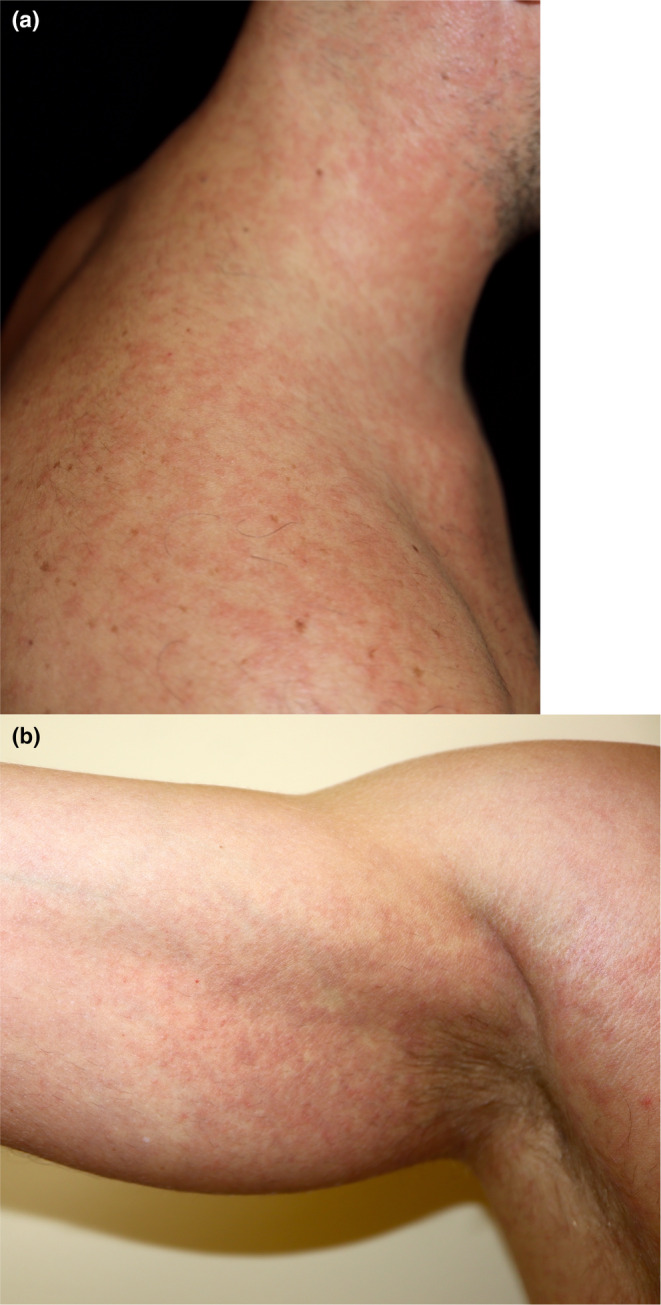
(a) Maculopapular rash in a patient with MPOX with neck and shoulder involvement. (b) Morbilliform rash in another patient in the right axilla.

Between 6% and 13.7% of patients with MPOX present with a confluent maculopapular rash on the trunk and proximal extremities concurrent with the primary MPOX lesions^129^ (Figure 5), but up to 17% of patients do not present with the typical pseudopustules,^130^ which complicates the diagnosis. About half of affected patients present with MPOX pharyngitis and some with associated Forchheimer spots. Pruritus may or may not be present. The rash usually appears 3 to 11 days after the onset of the disease, although cases have been reported in which it occurs simultaneously.^131^ A viral cause has been postulated, but drug or concurrent infections have been proposed in some patients. The failure to detect these contributing factors in many cases gives strength to the theory of MPOX as the causative agent. Histology of these lesions shows a superficial lymphocytic infiltrate, consistent with a maculopapular exanthem of viral or drug‐induced origin.^132^, ^133^

**FIGURE 6:**
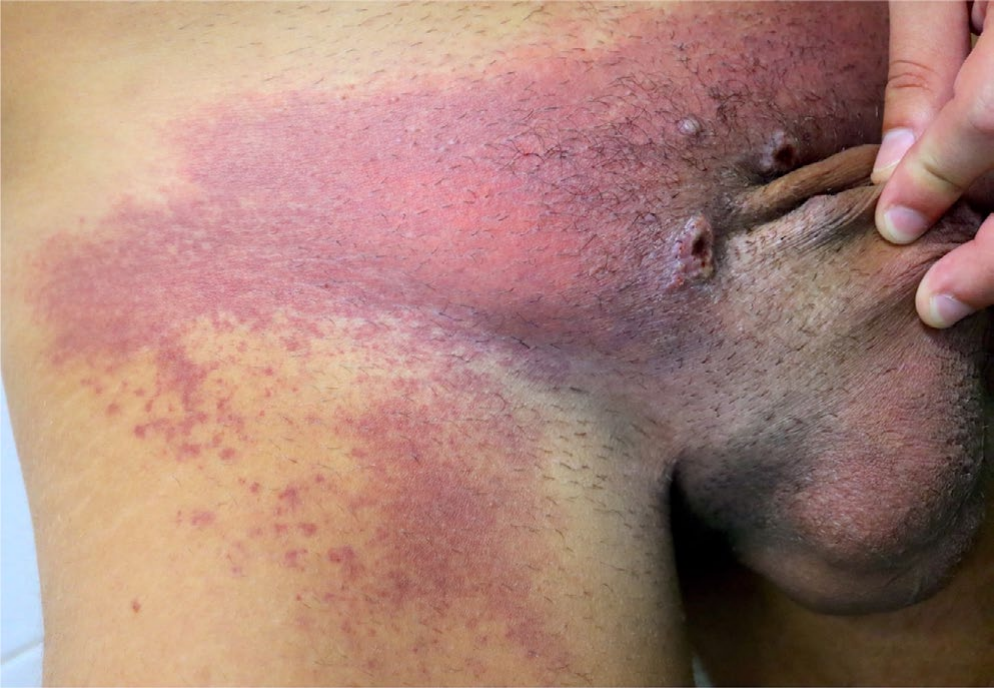
Erythematopurpuric plaque affecting the pubis and anterior thigh in a patient with multiple inflammatory pseudopustules on the penis.

A patient with hemorrhagic purpuric lesions in the inguinal region, who also had multiple perianal vesiculopustules and significant inguinal lymphadenopathy, has been reported (Figure 6). The authors propose that this phenomenon could be indirectly caused by the virus after negative results for viral RNA in the biopsy.^134^ A case of reactive panniculitis secondary to MPOX with a lupus panniculitis–like histology has recently been reported in a patient with recent MPOX infection.^135^

## 8 CONCLUSIONS

MPOX is a highly relevant and trending infection that has been declared a public health emergency of international concern twice in the past 2 years. It presents a characteristic clinical picture that has changed from the endemic cases described in Africa. With the explosive increase in the number of cases, we have witnessed multiple atypical manifestations that differ from the usual picture. It is crucial to be familiar with them, as they require a high degree of diagnostic suspicion and may involve different specialists.

## FUNDING INFORMATION

The authors declare that no funding sources were involved in the research, design, execution, or interpretation of this study.

## CONFLICT OF INTEREST STATEMENT

None declared.

## ETHICS STATEMENT

This study was conducted ethically in accordance with the principles outlined in the World Medical Association Declaration of Helsinki. Informed consent was obtained from the patient for being included in the study. All procedures, examinations, and handling of data were performed considering the patient's privacy and following ethical standards for medical research.

## Data Availability

The data that support the findings of this study are available from the corresponding author upon reasonable request.
